# Analysis and Prediction of Vehicle Kilometers Traveled: A Case Study in Spain

**DOI:** 10.3390/ijerph18168327

**Published:** 2021-08-06

**Authors:** Paúl Narváez-Villa, Blanca Arenas-Ramírez, José Mira, Francisco Aparicio-Izquierdo

**Affiliations:** 1 University Institute for Automobile Research Francisco Aparicio Izquierdo (INSIA-UPM), Universidad Politécnica de Madrid (UPM), 28006 Madrid, Spain; blanca.arenas@upm.es (B.A.-R.); francisco.aparicio@upm.es (F.A.-I.); 2 Transportation Engineering Research Group, Universidad Politécnica Salesiana, Cuenca 010105, Ecuador; 3 Statistics Department, Escuela Técnica Superior de Ingenieros Industriales (ETSII-UPM), Universidad Politécnica de Madrid (UPM), 28006 Madrid, Spain; josemanuel.mira@upm.es

**Keywords:** kilometers traveled, passenger vehicles, CART, random forest, gradient boosting, prediction, mobility pattern

## Abstract

Knowledge of the kilometers traveled by vehicles is essential in transport and road safety studies as an indicator of exposure and mobility. Its application in the determination of user risk indices in a disaggregated manner is of great interest to the scientific community and the authorities in charge of ensuring road safety on highways. This study used a sample of the data recorded during passenger vehicle inspections at Vehicle Technical Inspection stations and housed in a data warehouse managed by the General Directorate for Traffic of Spain. This study has three notable characteristics: (1) a novel data source is explored, (2) the methodology developed applies to other types of vehicles, with the level of disaggregation the data allows, and (3) pattern extraction and the estimate of mobility contribute to the continuous and necessary improvement of road safety indicators and are aligned with goal 3 (Good Health and Well-Being: Target 3.6) of The United Nations Sustainable Development Goals of the 2030 Agenda. An Operational Data Warehouse was created from the sample received, which helped in obtaining inference values for the kilometers traveled by Spanish fleet vehicles with a level of disaggregation that, to the knowledge of the authors, was unreachable with advanced statistical models. Three machine learning methods, CART, random forest, and gradient boosting, were optimized and compared based on the performance metrics of the models. The three methods identified the age, engine size, and tare weight of passenger vehicles as the factors with greatest influence on their travel patterns.

## 1. Introduction

Spain, similarly to other countries in Europe and the world, has intensified the application of policies aimed at the reduction of the number of road accidents and victims, and has been highly successful. The country has been among the lowest in ranking for the deaths per million inhabitants indicator for EU-28 countries since 2013 [1]. For exposure, an analogous indicator does not exist in Europe since the denominator (exposure) is generally a quantity estimated globally and is difficult to obtain if it is related to values from groups of interest.

The achievement of new road safety goals requires specific measures aimed at areas and groups with different characteristics, and this creates the need to improve knowledge on the real risk levels of user groups, defined by gender or age criteria, and of types and construction characteristics of vehicles, performance, and effectiveness of security systems, among other factors. This more disaggregated analysis approach faces the problem and difficulty of having data available to assess the real exposure levels of the specific groups as a starting point for the adoption of more appropriate measures for each situation.

In road safety analysis, three dimensions are considered: exposure, accident risk, and loss [2,3]; where accident risk is determined by the ratio between the number of accidents and exposure. The DRAG (Demande Routière, Accidents et Gravité) methodology has developed models in a multi-layer structure integrating the three main road safety dimensions (exposure, accident frequency, and severity). It was first used by Gaudry [4] for the province of Quebec under the name DRAG-1; it was also applied in the study of accidents on Spain’s interurban network [5] and specifically in vans [6]; in Algeria it was used in the construction of a first country-wide model of demand for road use and of road safety outcomes [7].

In practice, “exposure” refers to distance travelled, time spent travelling, or number of vehicles present on the road, and these measures are of the utmost importance when determining if a given driver group (by age, vehicle in use, gender, etc.) suffers a higher proportion of road crashes or is more prone to injury than others, accounting for differences in crash risk and in exposure to risk [8,9]. The groups with a high number of crashes per year are identified from data extracted from the crash databases maintained by official agencies or departments, such as DGT in Spain.

Despite their importance, the exposure data are far from perfect due to the difficulties in acquisition and availability, particularly in the more specific (restricted) groups of drivers or with several risk combinations [10,11]. This is especially an issue in countries such as Spain, where the collection of the necessary information (for example, mobility surveys) for the direct determination of exposure is not carried out on a regular basis. Thus, studies related to accident risks in different locations, such as Spain [12,13,14,15], Kentucky [16], Queensland [17], and France [18], used quasi-induced exposure, which is developed solely from the accident data themselves [19], although it implies that the underlying assumptions are not explicitly validated before the exposure measurement is adopted [20]; responsibility assignment studies [21,22] and other works reviewed by Jiang [23] have used quasi-induced exposure.

It is clear that the risk factors of some groups of users or vehicles cannot be assessed if the accident data cannot be related to the exposure of the members of those groups, expressed, for example, in vehicle-km or person-km traveled, since this value is not available in databases and is not easy to estimate.

In 2014, a group of researchers from the University Institute of Automobile Research Francisco Aparicio Izquierdo (INSIA-UPM, for its initials in Spanish) developed a methodological approach to analyze the data recorded in the Vehicle Technical Inspection (ITV, for its initials in Spanish) Centers and used it to infer the mobility from a small sample of articulated buses or coaches in the framework of a research project for the General Directorate for Traffic of Spain (DGT) [24,25,26,27]. In 2017, the DGT published a brief analysis of the relationship of kilometers traveled with vehicle age and type.

The general objective of this study was to determine the exposure of passenger cars, measured by the number of Vehicle Kilometers Traveled per year (VKT), with the degree of disaggregation allowed by the data provided by the DGT and collected in ITV centers, and detect possible differences in the mobility of passenger cars, providing valuable information for applications in road safety studies. 

Since the preparation of the data for the models is a very important task, the criteria for cleaning the raw data when creating “clean” databases are presented here. This study applied models based on supervised Machine Learning techniques: Classification and Regression Trees (CARTs), Random Forest (RF), and Gradient Boosting model (GBM), for the prediction and uncertainty levels of the VKT by passenger vehicles in Spain.

This article is organized as follows: the second section reviews the state-of-the-art of the application of kilometers traveled by vehicles as a measure of exposure and the applications to the data collected in ITV centers. The third section explains the methodology applied in the development of this study. The fourth section presents and discusses the results. Lastly, the conclusions are presented.

## 2. Literature Review

The number of vehicle kilometers traveled is a key indicator with direct applications, such as in estimating mobility levels, understanding vehicle use, and establishing its influence on accident rates and the environment. However, in addition to its application to accident and environmental studies, it extends to the areas of sustainable development and quality of life. Other fields of application include the elaboration of regional, national, and international policies, infrastructure management and urban planning, traffic and transport management, and land use planning [28].

The kilometers traveled can be estimated with methods based on traffic measurements, such as odometer readings and traffic density measurements, as well as with methods not based on traffic measurements, such as household/driver surveys and fuel sales [29]. However, it should be noted that, in practice, the availability and level of disaggregation of kilometers per person and vehicle can vary significantly and depends highly on the type and characteristics of the data collection method [30].

The odometer reading method has the advantage of accurate records but some disadvantages: a very intensive use of resources; the possibility of erroneous readings, annotations, transcriptions, and alteration of odometers; inspected vehicles may be abandoned or deregistered, which reduces the sample size and the number of observations. In addition, this method does not allow association between geographic data and the travel variables measured [29].

A source of growing interest is the records from ITV centers, which have the odometer readings and can also provide important additional information regarding use, property, location, performance, and breakdowns, among others. Thus, the information collected in inspection centers can be used by researchers in the transport and road safety field.

In the study of traffic accidents, the number of kilometers per person or vehicle is probably the most frequently preferred measure of exposure, with the practical advantage that, in theory, it is available at the desirable level of disaggregation. However, in practice, this is difficult but can be significantly improved by taking advantage of additional data sources such as odometer readings recorded in ITVs [30]. Considering that the improvement of vehicle safety is among the objectives of ITV implementation, it can be evaluated by combining the data recorded in ITVs, with breakdowns and accident records [31,32,33]. In addition, ITV records can be used to search for mobility patterns, in relation to kilometers traveled and vehicle age [34], or the differences between travel patterns, depending on rural and urban areas and the dependence on vehicle age [35]. It is even possible to establish relationships between the kilometers traveled and the frequency of accidents involving drivers of different ages [36].

The kilometers traveled are applied in the analysis of the ecological properties of the vehicles, through the life cycle assessment method, considering the relationship between the vehicle’s mileage and its failure rate [37]. The study of gas emissions is becoming increasingly important in the environmental and quality-of-life area due to the impact on health and the environment. In the study of greenhouse gases, the number of kilometers is used to estimate CO_2_ emissions of the total vehicle population [38] and to obtain future perspectives [34]. In addition, CO, NOx, PM, and VOC emissions by vehicle category can be estimated [39] and, depending on the available information, their evolution over time can be analyzed by geographical area [40]. In addition to the problems caused by emissions of polluting gases, relationships have been found between kilometers traveled and the risk of being overweight or obese for segments of the population [41]. ITV records allow the study of the relationships between vehicle age, engine size, fuel type, and kilometers traveled, among others, as well as the probability of failing the gas emissions test [42,43,44]. Moreover, it is possible to determine the relationship between vehicle age and the noise levels emitted [45]. These studies make it possible to identify the most polluting vehicle groups and their characteristics, for which the kilometers traveled must be accurately estimated data, allowing environmental researchers or air quality administrations to understand the real situation of vehicle use and to evaluate air pollution control policies [46].

The studies of pollutant emissions and accidents provide information for the recommendation of vehicle inspection policies, which makes it possible to evaluate time intervals between inspections [47] or determine the conditions to deregister the oldest vehicles [48]. The records of the ITVs, which in theory should be compiled at yearly intervals (depending on vehicle age according to the regulations), could lead to proposals for the optimization of the intervals between inspections, in shorter or specific times, according to the use patterns of the vehicles [49,50].

The studies of infrastructure management, urban planning, and land use management also benefit from the knowledge of the kilometers traveled by the vehicle, since it can be used to establish the relationship between travel behavior and built-in environmental factors [51], and how this relationship can influence the choice of place of residence [52,53,54]. In addition, the kilometers traveled help evaluate urban models, such as “transit oriented development” and “Park and Drive” [55], that seek to reduce dependence on private vehicles. The congestion relief strategy through the increase in road capacity can be evaluated by analyzing the effect on the kilometers traveled [56].

As a result of the literature review, it was identified that the survey method has been used to obtain data [36,38,39,40,41,46,51,52,53,54,55,56] and is potentially subject to bias [35,49]. Its massive application to road safety studies in practice becomes impossible and economically unfeasible, limited by the volume and geographical origin of the same [34]. In other studies [31,32,33,42,43,44,45,47,48,49], the data have been obtained from the ITV centers, presenting as advantages the possibility of matching with other data sources (accident records) [31,32] and the follow-up of individual vehicles through the ITV test history [35], although this depends on the good quality of the data, and reliable data cannot be obtained for years prior to the implementation of mandatory ITV [33]. It has also been found that the information from ITV records is fragmented in local jurisdictions, limiting the geographical scope of the studies; on the other hand, the data obtained in ITVs have been used in accident and emission studies but not for mobility estimation.

In Spain, as in other countries, ITV records are stored for the whole country, but have not been exploited as a source of data in mobility studies. This motivated the development of a methodology in the present work that shows the importance and applicability of ITV data, with satisfactory results in the estimation of mobility. In addition, this study recommends the improvement of the collection process with complete and systematic records of the data and the integration of the records of the jurisdictions that have not been integrated at present.

## 3. Materials and Methods

### 3.1. Methodology: Flow Diagram

**Figure 1:**
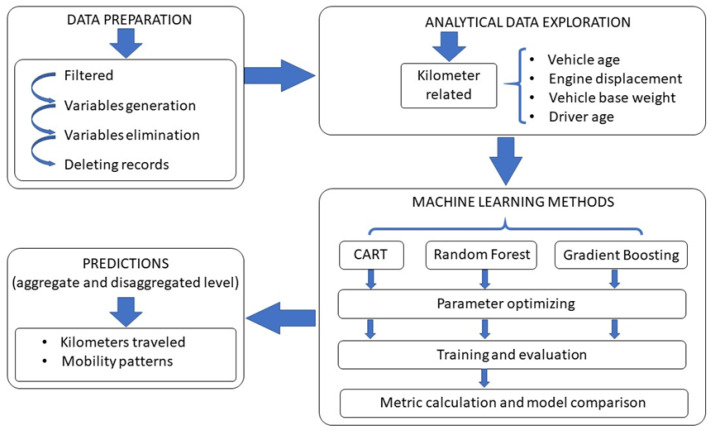
Analysis and prediction methodology.

Figure 1 shows the four-stage methodology applied in this study: Stage 1: data preparation, Stage 2: analytical data exploration, Stage 3: construction of selected Machine Learning models, and Stage 4: predictions. The methodology is described in detail below.

The sample of passenger vehicles was processed and filtered to create an Operational Data Warehouse (ODW), for the estimation and prediction of kilometers traveled by vehicles in Spain using advanced statistical models. The analytical exploration of the data was carried out considering a univariate and bivariate analysis of the ODW data. CART, Random Forest, and Gradient Boosting models were fitted for the selection of influence variables. The three models were compared based on performance metrics of predictive accuracy: Mean Squared Error (MSE), Root Mean Squared Error (RMSE), Mean Absolute Error (MAE), Mean Absolute Percentage Error (MAPE), and Coefficient of Determination (R^2^).

### 3.2. Data Preparation

This stage required the application of raw data filtering techniques and the generation of new variables of interest, as well as the elimination of variables and records according to the criteria described for each procedure. 

#### 3.2.1. Raw Data

In Spain, the data of distances traveled by the fleet vehicles in different periods are collected in the ITV files, and since 2011, the communication of these records to the DGT is mandatory. In addition, from 2013 the DGT vehicle registrations are transmitted telematically to all ITV stations.

The data used in this study were provided by the DGT and consist of 6,290,653 records of technical inspection tests carried out on passenger vehicles in the period 1985–2015 and handled in accordance with privacy policies. In addition to the pass or fail result of the test, each record contains data regarding: vehicle identification, technical data, ownership, inspection history, and defects history. Table 1 shows the 36 variables included in the database provided, as well as the percentage of invalid data. It is observed that there are variables with a high percentage of invalid data, which provide a perspective of the possible research applications to Spanish fleet vehicles given comprehensive information with objectives different from those of this study in the future.

**Figure 2:**
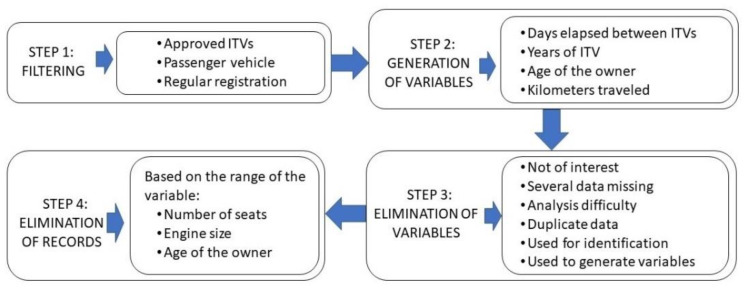
Data processing methodology.

The records of the information provided by the DGT were subject to a processing that consists of a four-step methodology: (1) filtering, (2) generation of variables, (3) elimination of variables, and (4) elimination of records. Figure 2 shows a summary of the processing methodology described below.

Step 1: The filtering was performed using the information from variables CLAVE, COD_TIPO_OBV, and COD_CLASE_MAT, which only include the information that corresponds to approved inspections, passenger vehicles, and ordinary registration to be retained.

Step 2: Some variables of interest for the study are not explicitly found in the database but can be obtained from the present data. They are listed below:

- Periodicity: this variable indicates the days elapsed between two consecutive inspections; it is calculated from the difference between two consecutive values registered in variable FEC_INSPECCION (ITV date).
- Kilometers traveled (VKT): this variable is determined by (1) where the difference between the odometer reading of the first ITV (X_1_) and the second reading (X_2_) is divided by periodicity (Y); this result is multiplied by 365 to obtain the kilometers in annual terms.
  (1) $$\mathrm{KV}=\left(\frac{X_{2}-X_{1}}{Y}\right)\cdot 365$$
- Vehicle age: this variable indicates how old the vehicle is when the inspection is carried out; it is calculated from the difference between the values registered in variable FEC_INSPECCION (ITV date) and FEC_PRIM_MAT (date of first registration).
- Age of the driver: the value of this variable is determined by establishing the age of the owner of the vehicle, with the reasonable assumption that, for passenger vehicles, the owner is the driver. This variable is calculated from the difference between variable FEC_INSPECCION (ITV date) and FEC_NACIMIENTO (date of birth of the owner)

Step 3: The criteria followed for the elimination of variables are: those that are not considered of interest for the safety-related study, those with a high proportion of missing data, those that make the analysis difficult, those that provide duplicate information, those that contain codes that make it possible to identify the successive inspections, those used for the generation of new variables, and those not applicable to the study. Table 2 lists the eliminated variables grouped according to the six criteria adopted.

Step 4: When the data obtained up to this step were reviewed, anomalies were found in the values of the generated variables (negative values, values equal to zero, and inconsistent values). This was due to records with missing or null values and inconsistencies in the recorded values, such as the decrease in odometer readings over time, or because the inspection date was before the date of the first registration, etc. Reasonable value ranges were established, such that the records with values outside them were eliminated. For variable NUM_PLAZAS (number of seats), a four to nine range was established, based on the definition presented in Royal Legislative Decree 6/2015, of October 30, which approves the revised text of the Law on Traffic, Circulation of Motor Vehicles and Road Safety. For variable CILINDRADA, values between 850 and 6600 cc were considered, given that they are the smallest and largest engine sizes of passenger vehicles for sale in Spain. For variable age of the driver, only values over 18 years old were considered, which is the minimum age to obtain a driver’s license. For variable PERIODIOCITY, obtained in step 2, a maximum limit of four years was established, considering that, in Spain, it is the maximum before an ITV is required (new vehicles), and an upper limit of four years and a minimum of 60 days was established according to Spanish traffic legislation, the latter being the time available to fix the problems from an unfavorable ITV, and considering that in this period mileages are abnormally low.

#### 3.2.2. Numerical Summary of the Variables

The final ODW obtained contains the information for variables: engine size, number of seats, age of the driver, province, vehicle age and tare, which were considered as predictive variables in the development of the Machine Learning models for the estimation and prediction of mobility in terms of kilometers traveled. Table 3 shows the descriptive statistics of the predictor variables.

### 3.3. Analytical Data Exploration

Consecutive records of ITV tests can be used to explore how the annual VKT has evolved over time. The annual VKT evolution and its dependence on the vehicle attributes (vehicle age, engine size, age of the driver, and tare) is established at the vehicle population level. Each vehicle attribute has been segmented into ranges that were selected to coincide with those used by the DGT in the publication of statistics related to the fleet vehicles. Furthermore, it is possible to compare the evolution of the annual VKT between different years to unveil existent relationships between the variables and their evolution over time.

#### Univariate Data Analysis

**Figure 3:**
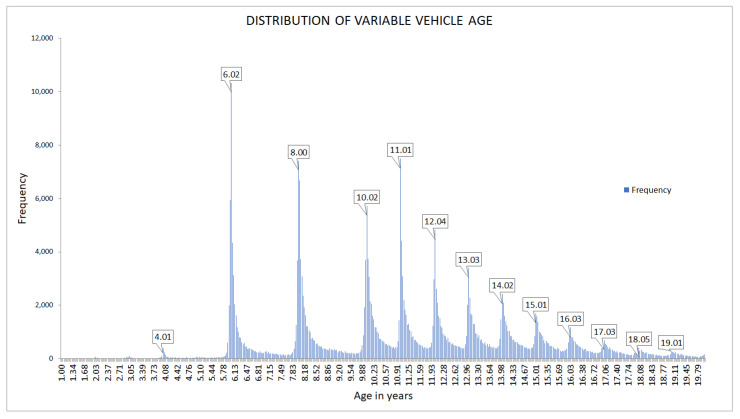
Distribution of variable vehicle age.

The analysis of the distribution of the data recorded for the vehicle age variable found several peaks, as observed in Figure 3. These peaks occur when the age of the vehicle is 4, 6, 8, and 10 years and from this point on, every year. This is interesting since it coincides with the age at which vehicles are required to go through their mandatory inspection in Spain, which shows that there is compliance with the regulations.

**Figure 4:**
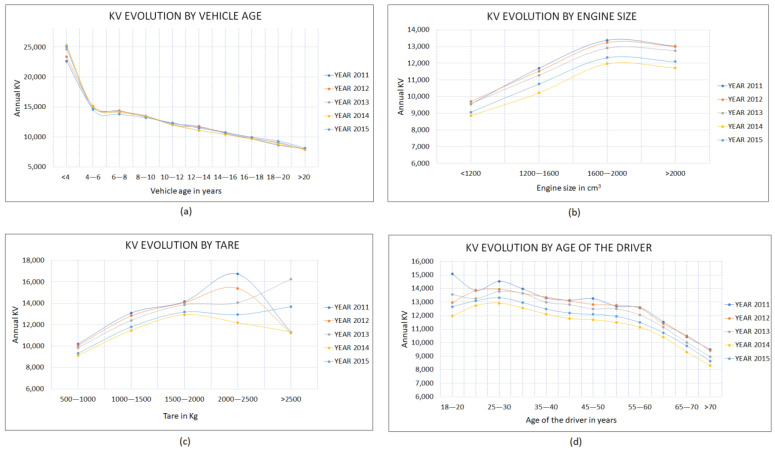
Distribution of kilometers per year by vehicle attribute. (**a**): KV evolution by vehicle age; (**b**): KV evolution by engine size; (**c**) KV evolution by tare; (**d**): KV evolution by age of the driver.

Figure 4 shows the distribution along time of kilometers traveled and the dependence on vehicle attributes; this establishes the following mobility patterns:

(1) The relationship between annual VKT and vehicle age shows similar behavior when the data of the five years studied are compared. It is observed that the annual VKT of the vehicles decreases as vehicle age grows, with an inflection point in the range of four to six years. Figure 4a shows two different behaviors in passenger vehicle mobility: one for vehicles up to six years old and another for those over six years old. The rate of mean VKT decline for newer vehicles is higher than for older vehicles. In addition, vehicles less than four years old have approximately twice the VKT of those that are in the 10 to 12 years range and approximately three times that of vehicles older than 20 years;
(2) Figure 4b shows that vehicles with engine size larger than 1600 cm^3^ have the highest VKT and are in approximately 30% better shape than those with engine size smaller than 1200 cm^3^, which have the lowest mean VKT value. This information is relevant and reveals a different mobility pattern depending on the composition of the passenger vehicle fleet in terms of engine size, considering that, according to the registration statistics published in DGT (2015), vehicles with an engine size in the range of 1200 to 1600 cm^3^ represent approximately 54% of the fleet and, if greater than 1600 cm^3^, approximately 27%;
(3) Vehicles with higher tare weight travel more VKT per year, as Figure 4c shows, which is logical considering that they tend to use engines with greater cubic capacity and higher loads in long routes;
(4) There is a reduction in mobility as the age of the driver increases, as Figure 4d shows. For ages in the range of 25 to 30 years, VKT values slightly higher than the rest are observed, and from ages in the range of 55 to 60 years, there is an increase in the rate at which VKT decline, traveling on average 1000 VKT less for every five-year increase.

**Figure 5:**
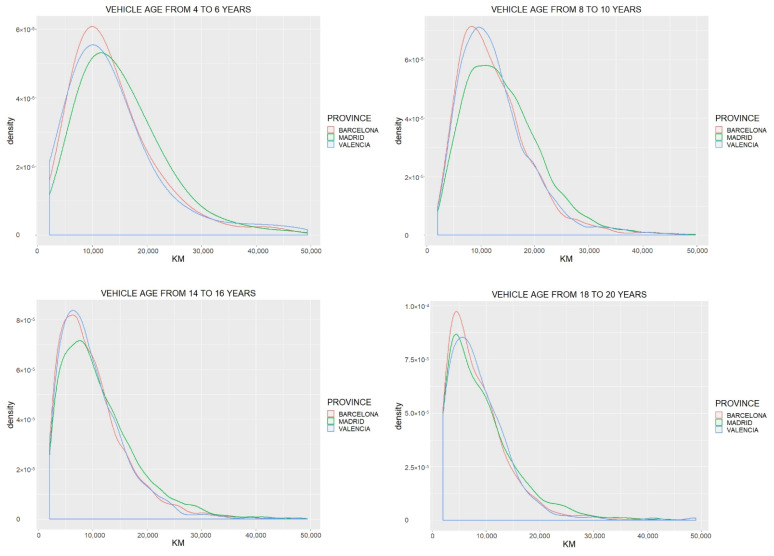
Comparison of kilometers per year for the provinces of Barcelona, Madrid, and Valencia, in relation to vehicle age.

At the total vehicle fleet level, a decrease in annual VKT with vehicle age was observed. This behavior is similar in the different provinces of Spain. The comparison was carried out through the distribution of annual VKT, of the different provinces, and in four vehicle age ranges. As an example, the provinces of Barcelona, Madrid, and Valencia were compared. The results are shown in Figure 5, where the shift of the distributions to the left indicates a decrease in kilometers as vehicle age increases; this behavior is consistent in all provinces. Differences in kilometers in the different provinces are also observed; however, as vehicle age increases, they tend to disappear, which shows that passenger vehicles in Spain behave similarly to those described in [13].

### 3.4. Machine Learning Methods (MLM)

#### 3.4.1. Classification and Regression Tree (CART)

In the area of transportation, the CART method has been applied to study the utility factors of plug-in hybrid electric vehicles [57], to explore causes and effects of automated vehicle disengagement [58], and in the development of models for vehicular traffic noise prediction [59]. It has also been widely used to study road safety, as shown in the summary presented by [60], which cites 14 studies related to traffic accidents.

Classification and regression trees (CARTs) are the traditional building blocks of data mining and the classic algorithm for Machine Learning. An advantage of this method is the simplicity of the resulting model, where the decision tree is very easy to understand and interpret [61]. Tree-based methods divide the space of inputs into a set of polytopes and then fit a simple model into each one [62]. In a regression problem, the observations with similar response values are split into the same region, and a constant value (mean) is predicted within each region. The appropriate variables and split points are selected by minimizing the mean square error (MSE) as the loss function. Once the loss function is minimized, the split variable and the split point can be selected [63]. 

In a regression problem, assuming that Y is the response variable predicted by inputs *p* (*x*1, *x*2*… xp*), the estimation resolution is carried out in four steps, as indicated in [63]:

1. Start with all the cases in a region, which is the root node.
2. At each internal node of the tree, a test is carried out on one of the predictors *xj*.
3. Depending on the test result, the observations are allotted to the left or right subregion (branch) of the tree.
4. Step 3 is repeated until reaching a terminal node or leaf in which a prediction is made.

The R software was used to develop the model considering the fitting of hyperparameters “cp”, “minsplit” and “maxdepth”, which are thoroughly described in [64] and summarized in Table 4.

#### 3.4.2. Random Forest (RF)

The Machine Learning Random Forest method, developed by Breiman, has been applied in several transport studies; [65] presents a summary of its application to studies of travel mode choice behavior, prediction of traffic incidents, and travel time and flow prediction, as well as pattern recognition. It has also been applied in the study of accidents, to identify patterns of accident frequency and severity [66], accident likelihood and severity [67], and precrash maneuvers [68]. The RF method has also been applied in the field of plug-in hybrid vehicles and autonomous vehicles to study utility factors [57] and to assess threats present in their operation, such as obstacles, pedestrians, and other vehicles [69].

The RF method is an ensemble of trees, such that each tree depends on the values of a random matrix sampled independently and with the same distribution for all trees in the forest [70]. In the RF environment, many classification and regression trees are built using randomly selected training data sets and random subsets of predictor variables to model results; in each split, only a randomly selected subset of the input variables is considered, as opposed to standard CART, where all input is taken into account. The results from each tree are aggregated to provide a prediction for each observation, which can be more accurate than a single decision tree model [71]. 

A summary of the construction procedure of the RF model is presented as follows [62]:

1. For *b* = 1 to *B*:
  - A size *N* Bootstrap Z* sample of the training data is drawn.
  - An RF tree is grown to the bootstrapped data, recursively repeating the following steps for each node of the tree, until the minimum node n_min_ is reached.
    - Select m variables randomly from the p variables;
    - Choose the best variable/split point among m;
    - Split the node into two child nodes.
2. Exit the set of trees.

The R software was used to develop the model, considering the fitting of hyperparameters “num.trees”, “mtry”, “min.node.size”, and ”sample.fraction”, which are thoroughly described in [72] and summarized in Table 4.

#### 3.4.3. Gradient Boosting Model (GBM)

In the transport area, methods based on the boosting model have been applied to the study of road characteristics [73] and environmental conditions [74] associated with the occurrence of traffic accidents, in addition to the severity of the injuries produced [75,76]. Reference [77] analyzes the effects of driving behavior (characteristics of the driver and the vehicle) on the level of polluting gases from the vehicles.

The GBM is an additive model that involves the sequential combination of a large number of trees or estimators in a single composite model, adding the simple trees one at a time without changing the data in the model; specifically, a repeated sampling is not used. In this model, with each estimator added, the largest errors of the previous estimator are corrected, and gradient descent is used to optimize the loss function.

For a regression model, the GBM algorithm works as follows [78]:

1. Select tree depth, D, and the number of iterations, K;
2. Compute the average response, ӯ, and use this as the initial predicted value for each sample;
3. For *k* = 1 to K:
  - Compute the residuals, the difference between the observed value and the current predicted value for each sample;
  - Fit a regression tree of depth *D* using the residuals as the response;
  - Predict each sample using the regression tree fit in the previous step;
  - Update the predicted value of each sample by adding the previous iteration’s predicted value to the predicted value generated in the previous step.
4. The process ends.

The R software was used to develop the model, considering the fit of hyperparameters: “n.trees”, “interaction.depth”, “n.minobsinnode”, “shrinkage”, and “bag.fraction”, which are thoroughly described in [79] and summarized in Table 4.

#### 3.4.4. Performance Metrics for Model Comparison

Metrics applied to a set of continuous values were used to evaluate the predictions made with the regression models. The evaluation metrics used are Mean Squared Error (MSE), Root Mean Squared Error (RMSE), Mean Absolute Error (MAE), Mean Absolute Percentage Error (MAPE), and Coefficient of Determination (R^2^).

The MSE calculates the average of the squared difference between actual values (y_i_) and the predictions made (ŷ_i_); this is computed by (2).

(2) $$\mathrm{MSE}=\frac{1}{N}\overset{N}{\underset{i=1}{\sum }}{\left(y_{i}-{\hat{y}}_{i}\right)}^{2}$$

The RMSE is calculated by obtaining the square root of the MSE; this is performed to ensure that the scale of the errors coincides with the scale of the response variable, which is computed by (3).

(3) $$\mathrm{RMSE}=\sqrt{\frac{1}{N}\overset{N}{\underset{i=1}{\sum }}{\left(y_{i}-{\hat{y}}_{i}\right)}^{2}}$$

The MAE calculates the average absolute distance between prediction values (ŷ_i_) and actual values (y_i_); the MAE is computed by (4).

(4) $$\mathrm{MAE}=\frac{1}{N}\overset{N}{\underset{i=1}{\sum }}\left|y_{i}-{\hat{y}}_{j}\right|$$

The MAPE values are expressed as a percentage which facilitates conceptualization. The MAPE metric is robust in the presence of outliers due to the use of the mean value in the denominator; MAPE is computed by (5).

(5) $$\mathrm{MAPE}=\frac{100\%}{N}\overset{N}{\underset{i=1}{\sum }}\left|\frac{y_{i}-{\hat{y}}_{j}}{y_{i}}\right|$$

The Coefficient of Determination R^2^ is an evaluation metric closely related to MSE and has the advantage of being scale-invariant. R^2^ is determined by (6).

(6) $$R^{2}=1-\frac{\mathrm{MSE}\left(\mathrm{model}\right)}{\mathrm{MSE}\left(\mathrm{baseline}\right)}$$

To calculate the MSE (model), (2) is applied and the MSE (baseline) calculates the average of the squared difference between actual values (y_i_) and the mean of y_i_ (represented by ӯ); the MSE (baseline) is computed by (7).

(7) $$\mathrm{MSE}\left(\mathrm{baseline}\right)=\frac{1}{N}\overset{N}{\underset{i=1}{\sum }}{\left(y_{i}-\bar{y}\right)}^{2}$$

## 4. Results

The selected models were compared based on the fit obtained, after the hyperparameters of each of each model were optimized, and on the prediction errors. The models were also compared in their ability to determine the importance of variables relevant to the estimation and inference of the mobility. The GBM models were used to extract complex patterns from the data.

### 4.1. Parameter Optimization

The values of several hyperparameters were optimized to improve the predictive capacity of the models and facilitate their training. To find the optimal values, a grid search algorithm was used to search automatically in a series of models adjusted with iterations of combinations of hyperparameter values; this evaluated the combination and hyperparameter values that work best with the minimum error value criterion (RMSE). Table 4 shows the values obtained with the optimization process and used in the different models.

### 4.2. Performance of Prediction Models

The database was divided into two sets: one for the training data, which the algorithm uses for learning, and the other for the test data, used to measure and compare the accuracy of the models. To find the best split strategy, two proportions were used (80–20% and 70–30%). Training, which the algorithm uses for estimation, and the other test data were chosen by stratified sampling to help the response variable achieve a balanced distribution in both data sets. Each model was executed 20 times, and the results showed consistency. The performance and comparison of the regression models were carried out using the RMSE, MAE, R^2^, and MAPE metrics. Table 5 shows the average results of the metrics obtained; the results show that there are no significant differences with different training and test data proportions used in the models, and it is observed that the 80–20% proportion performs slightly better, as outlined in the following results.

### 4.3. Prediction and Errors

**Figure 6:**
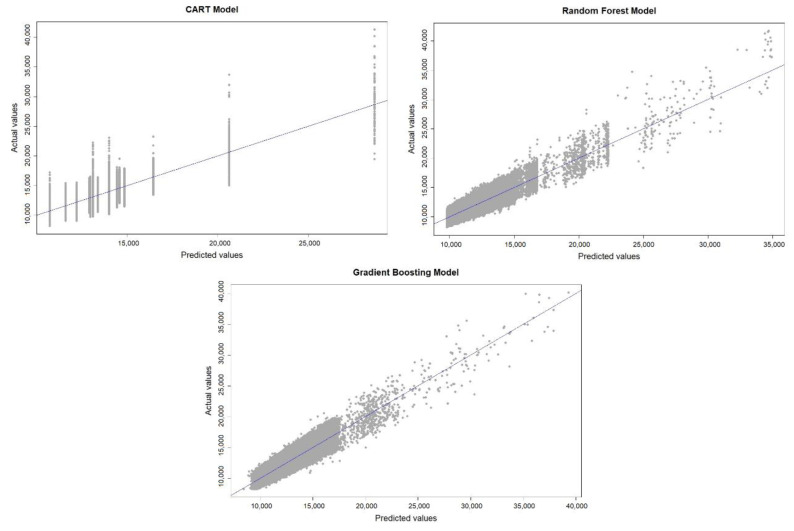
Comparison of predictions and actual values with the CART, RF, and GBM models.

Figure 6 shows the scatter plot of the predicted and actual values with the application of the CART, RF, and GBM models, where the coincidence of points with the line means that the predicted value is equal to the actual one. The error produced in the prediction is interpreted based on the distance that separates the points from the line. The GBM shows a more uniform distribution of points on both sides of the line, which indicates a lack of prediction bias and that it outperforms the CART and RF models. Thus, the GBM has a better performance prediction, which is confirmed when the values of the metrics between the different models are compared (Table 5), where the GBM has a higher R^2^ value (0.748) and lower RSME (1220.328), MAE (1035.395), and MAPE (0.748) values. In addition, the best predictions are obtained below a VKT of approximately 22,000 km; beyond this point, there is a small increase in the spread of the predicted values.

### 4.4. Variable Importance

**Figure 7:**
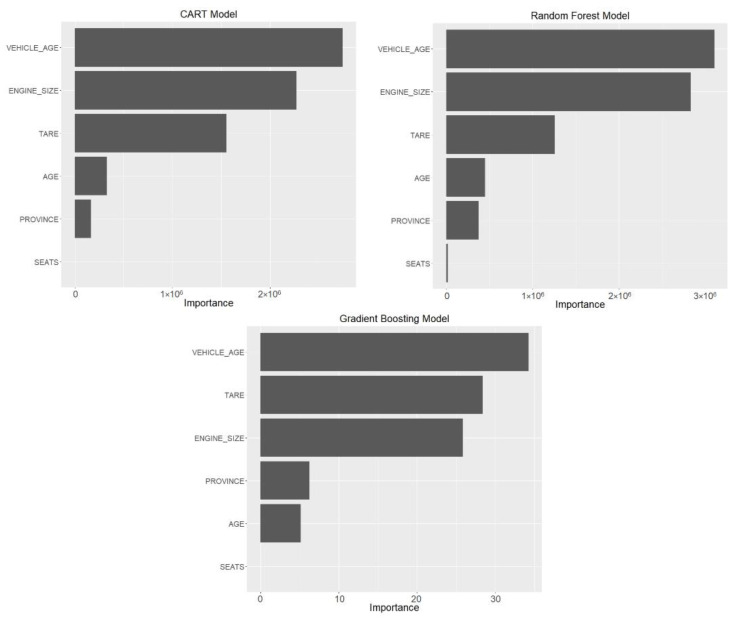
Importance of the variables.

To interpret how the model prediction process functions, it is appropriate to assess the importance of the variables, which is established by the permutation method, in which the reduction in prediction accuracy is measured by randomly permuting the variables. Figure 7 illustrates the importance established by the different models. The three models select vehicle age as the most important variable in the prediction of mobility, followed by engine size and tare weight, which shows that vehicle performance also has a great influence on mobility. Furthermore, the age of the driver and province have less importance, and the number of vehicle seats has practically no influence on mobility.

### 4.5. Relevant Pattern Recognition with Selected Machine Learning Models

**Figure 8:**
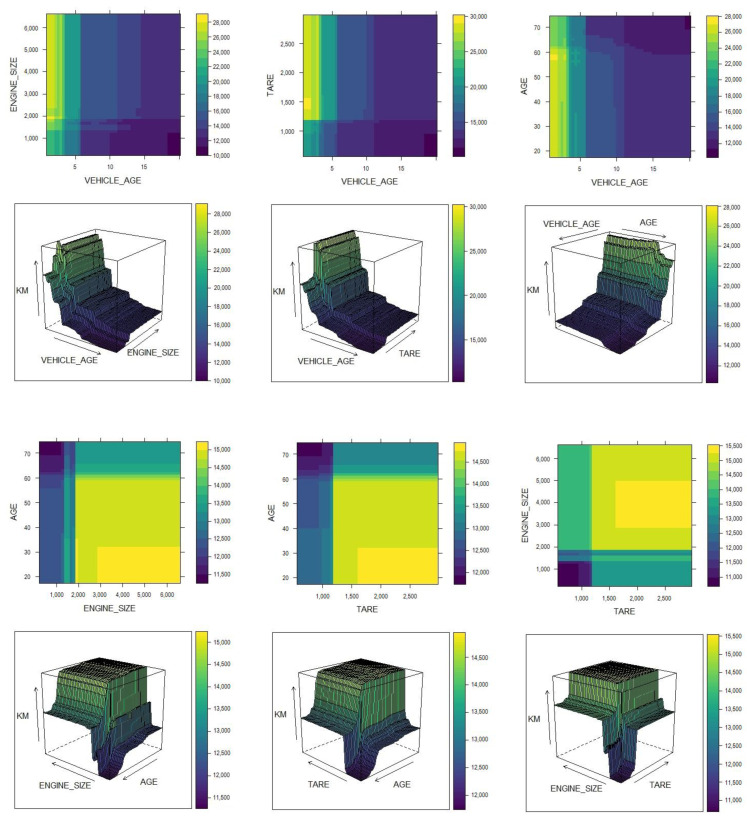
Partial dependence graph for the GBM.

In addition to achieving good results in the prediction of mobility, it is important to understand how the variables interact or relate to each other in order to determine the prediction. To this end, and based on the GBM, the partial dependence graphs in Figure 8 were obtained to show the dependence of VKT values on pairs of variables that were selected as the most important based on Figure 7.

Figure 8 shows a sharp drop in VKT up to a vehicle age of approximately five years, after which the decrease in VKT is smooth, with less noticeable changes. This behavior is constant for all values of engine size, tare weight, and age of the driver; however, vehicles with engine sizes larger than ≈2000 cm^3^, a tare weight greater than ≈1200 kg, and drivers aged less than ≈60 years have higher VKT values. The partial dependence of the VKT on the age of the driver/engine size and age of the driver/tare weight shows that drivers under ≈60 years old have higher VKT when they use vehicles above ≈2000 cm^3^ or when the vehicle weighs more than ≈1200 kg; in both cases, a VKT increase of ≈20% is observed. When considering the engine size and tare weight variables, it is observed again that vehicles that combine values greater than ≈2000 cm^3^ and ≈1200 kg have higher VKT. The information provided by the GBM is consistent with the analysis outlined in Section 3.3.

As an example of the application of the models and based on the patterns identified, Table 6 shows the VKT values predicted with the RF and GBM models for different values of the input variables. It also shows the uncertainty intervals estimated with the RF model, which is composed of individual decision trees and therefore can estimate each individual Random Forest tree and determine the bounds; based on this, the confidence interval of this example is 95%. The values in the predictions with the RF and GBM models are similar and consistent with the behavior patterns found.

## 5. Discussion

The application of the knowledge of more realistic exposure levels of vehicles classified or grouped by characteristics of interest is of great relevance in accident research. This is possible due to the exploitation of the data that ITV centers register when technical inspections are performed on vehicles. The information from ITV centers has not been used before in comprehensive mobility studies and, after an adequate preparation process, has shown enormous potential for exploitation since it opens up the possibility of replicating this study with other types of Spanish fleet vehicles, such as vans, trucks, coaches, motorcycles, etc. Its potential lies in that a single source consolidates the information on vehicle make/model, vehicle performance (fuel consumption, power, and weight/power ratio, among others), as well as polluting emissions (gases and noise) and defect history, which can be applied for other research purposes.,

This study shows satisfactory results in the estimation of the mobility of passenger vehicles, as measured by the VKT, considering the values of the performance metrics of the models, RMSE ≈ 1200, MAE ≈ 1100, and R^2^ and MAPE ≈ 0.7. The results obtained at a disaggregated level can be considered a measure of the exposure of passenger vehicles in Spain, for which the three models developed (Figure 7) have found that mobility is mainly determined by vehicle age, engine size, tare, age of the driver, and, to a lesser extent, the province and number of seats. 

Depending on the variable for which the behavior pattern needs to be predicted or understood, additional information and new variables can be incorporated, since the models implemented in the methodology developed in this study have flexibility to be adapted and used according to the analysis needed.

## 6. Conclusions

The data preparation methodology applied to the records of ITV centers made it possible to establish an appropriate database for use in mobility analyses through the VKT of passenger vehicles in Spain.

Through an analytical exploration of the data, some mobility patterns were established in relation to vehicle age, engine size, tare weight, age of the driver, province, and number of seats. The patterns identified are consistent with the partial dependence results, subsequently obtained with the Gradient Boosting model.

This study used three Machine Learning models: CART, Random Forest, and Gradient Boosting. The models were optimized by determining the best values for different hyperparameters used in the estimation. The evaluation of the models through the metrics RMSE, MAE, R^2^, and MAPE indicates that Gradient Boosting has the best prediction performance.

The three models make it possible to establish that, for passenger cars, vehicle age is the most important factor in mobility, followed by those related to the characteristics of the vehicle (engine size and tare weight) and the age of the driver. The variable that characterizes territorial mobility (the province variable) is the least important and may indicate that geographically distributed mobility does not show significant differences, which is also the case for the variable number of seats. The partial dependence analysis performed with the Gradient Boosting model complements the understanding of the influence of the different variables on mobility.

Although the models developed have allowed a disaggregated mobility study, the level of disaggregation has been limited to using six input variables for the models, based on data availability. In future work and with an update of the data, it is of interest to carry out a more in-depth study of mobility with the inclusion of new variables in the models, such as: engine power, CO_2_ emissions, fuel used, fuel consumption, and history of defects, all of them recorded in the passage of vehicles through the ITV. The methodology developed in the present work is feasible for application to other types of vehicles of interest, such as buses, motorcycles, and trucks. In addition to having a refined database, there is the potential for future work using a cross-reference of information with databases of accidents and drivers.

In-depth knowledge of the reality of mobility can be used as a very important resource for the proposal, monitoring, and revision of policies and regulations in areas related, for example, to road safety (risk indicators, driver behavior), air quality (emissions), and energy consumption (tourism vehicles, cargo vehicles, vehicle fleets, etc.).

## Figures and Tables

**Table 1:** Variables of the data provided by the DGT.

Field	Variables: ITV Code (Description)	No. of Records with Zero Value	No. of Empty Records	Percentage of Invalid Records ^1^
Vehicle identification	newid (Vehicle Identification Code)	0	0	0.00%
	FEC_MATRICULA (Date of registration)	0	0	0.00%
	COD_CLASE_MAT ^2^ (Registration class)	6,258,376	0	99.49%
	JEFATURA_MAT_NORM (Province of registration)	0	0	0.00%
	COD_MARCA_OBV (Vehicle Make Identification)	0	0	0.00%
	MODELO_OBV (Model description)	0	218	0.00%
	COD_TIPO_OBV (Vehicle type)	0	0	0.00%
	FEC_PRIM_MAT (Date of first registration)	0	0	0.00%
	RENTING (Rental vehicle)	0	4,878,481	77.55%
Technical data	CO_2_ (CO_2_ emissions)	8135	6,161,708	98.08%
	TIPO_ALIMENTACION (Fuel type)	2	6,268,947	99.65%
	CILINDRADA_OBV (Engine size)	2481	0	0.04%
	POTENCIA_OBV (Tax horsepower of the vehicle)	3207	0	0.05%
	TARA_OBV (Tare weight)	2529	0	0.04%
	PESO_MAX_OBV (Maximum weight)	47,563	0	0.76%
	NUM_PLAZAS_MAX (Maximum number of seats)	2667	0	0.04%
	MTMA (Maximum Technically Permissible Mass)	5,114,835	0	81.31%
	MMC (Mass in running order)	5,079,130	0	80.74%
	KW (Maximum net power)	5,199,797	0	82.66%
	RPP (Weight power ratio)	6,267,254	0	99.63%
	CARROCERIA (Bodywork type)	0	6,272,460	99.71%
	CONSUMO (Fuel consumption)	6,290,653	0	100.00%
	DISTANCIA_EJES (Wheelbase)	6,271,015	0	99.69%
	CODIGO_ECO (Eco code)	0	6,290,664	100.00%
	CATELECT (Electric vehicle)	0	6,285,655	99.92%
	AUTELECT (Electric vehicle range)	4494	6,286,140	100.00%
Ownership	FEC_NACIMIENTO (Date of birth of the owner)	0	356,010	5.66%
	PERSONA_JURIDICA (Legal entity)	0	5,933,259	94.32%
Technical inspection history	FEC_INSPECCION (ITV date)	0	844,837	13.43%
	NUM_ITV (Technical inspection number)	0	0	0.00%
	CLAVE (Vehicle technical inspection result)	0	0	0.00%
	COD_PROVINCIA (Province of domicile of the vehicle)	0	80	0.00%
	KM1 (Odometer reading)	0	3,954,130	62.86%
History of defects	DESC_GRUPO_DEFECTO_1 (Breakdown location group)	0	5,623,957	89.40%
	DESC_DEFECTO_1 (Breakdown location element)	0	5,623,957	89.40%
	COD_CALIFICACION_DEF_1 (Breakdown severity)	0	5,603,109	89.07%

^1^ Includes missing values and values equal to zero. ^2^ Not considered an invalid variable since the value of zero corresponds to a registration type category.

**Table 2:** Variables eliminated.

Variable Elimination Criteria	Variables: ITV Code	Description
Not considered of interest	PERSONA_JURIDICA	Legal entity
	DESC_GRUPO_DEFECTO_1	Breakdown location group
	DESC_DEFECTO_1	Breakdown location element
	COD_CALIFICACION_DEF_1	Breakdown severity
High proportion of missing data	RENTING	Rental vehicle
	CO_2_	CO_2_ emissions
	TIPO_ALIMENTACION	Fuel type
	MTMA	Maximum Technically Permissible Mass
	MMC	Mass in running order
	KW	Maximum net power
	RPP	Weight power ratio
	CARROCERIA	Bodywork type
	CONSUMO	Fuel consumption
	DISTANCIA_EJES	Wheelbase
	CODIGO_ECO	Eco Code
	CATELECT	Electric vehicle category
Analysis difficulty ^1^	COD_MARCA_OBV	Make Identification
	MODELO_OBV	Model description
Duplicate information	FEC_MATRICULA	Date of registration
	JEFATURA_MAT_NORM	Province of registration
Used for identification	newid	Vehicle Identification Code
	COD_CLASE_MAT	Registration class
	CLAVE	Vehicle technical inspection result
	COD_TIPO_OBV	Vehicle type
Used to generate new variables	FEC_PRIM_MAT	Date of first registration
	FEC_INSPECCION	ITV date
	FEC_NACIMIENTO	Date of birth of the owner
	KM1	Odometer reading

^1^ The information to relate database codes with the make or model of the passenger vehicle is not available.

**Table 3:** Predictive variables of kilometers per year.

Variable	Description	Min	Max	Mean	S.D.
Engine size	Engine size	852	6292	1765	384.12
Seats	Occupant capacity (discrete variable)	4	9	NA	NA
Age	Age of the driver	18	80	60.37	13.51
Province	Province of registration (categorical variable)	NA	NA	NA	NA
Vehicle age	Vehicle age	1	39.96	12.37	4.20
Tare	Vehicle tare weight	620	2960	1219	224.85

**Table 4:** Hyperparameters used in the execution of the models.

Model	Hyperparameters Description	Value
CART	cp: Complexity parameter.	0.01
	minsplit: The minimum number of observations that must exist in a node in order for a split to be attempted.	5
	maxdepth: Set the maximum depth of any node of the final tree, with the root node counted as depth 0.	17
Random Forest	num.trees: Number of trees to grow.	200
	mtry: Number of variables randomly sampled as candidates at each split.	5
	min.node.size: Minimal node size.	10
	sample.fraction: Fraction of observations to sample.	0.5
Gradient Boosting	n.tress: Integer specifying the total number of trees to fit.	1998
	Interaction.depth: Integer specifying the maximum depth of each tree.	7
	n.minobsinnode: Integer specifying the minimum number of observations in the terminal nodes of the trees.	15
	shrinkage: a shrinkage parameter applied to each tree in the expansion.	0.1
	bag.fraction: the fraction of the training set observations randomly selected to propose the next tree in the expansion.	1

**Table 5:** Metrics comparison of the different regression models.

Model	Training–Test Proportion							
	80–20%				70–30%			
	RMSE ^1^	MAE ^1^	R^2^	MAPE	RMSE ^1^	MAE ^1^	R^2^	MAPE
CART	1397.604	1146.963	0.670	0.084	1396.622	1147.038	0.669	0.084
Random Forest	1232.291	1042.873	0.744	0.076	1233.614	1043.207	0.742	0.076
Gradient Boosting	1220.328	1035.394	0.748	0.075	1221.665	1036.148	0.747	0.075

^1^ Units are kilometers.

**Table 6:** Prediction examples.

	Example 1	Example 2	Example 3	Example 4	Example 5	Example 6
Vehicle age	3 years	4 years	8 years	8 years	15 years	15 years
Engine size	2500 cm^3^	1600 cm^3^	2500 cm^3^	1600 cm^3^	2500 cm^3^	1600 cm^3^
Tare	1500 kg	1000 kg	1500 kg	1000 kg	1500 kg	1000 kg
Age	65 years	40 years	65 years	40 years	65 years	40 years
Province	Madrid	Sevilla	Segovia	Valencia	Zaragoza	Barcelona
Seats	5	4	5	4	5	4
Lower bound ^1^	23,839	12,849	14,217	12,237	11,945	11,509
RF prediction	27,871	17,840	14,386	12,777	12,245	11,762
GBM prediction	25,008	17,384	14,415	14,084	12,210	11,499
Upper bound ^1^	30,846	20,820	14,599	14,526	12,416	11,975

^1^ 95% confidence interval bounds.

## Data Availability

Data sharing not applicable.
